# Gene diversity explains variation in biological features of insect killing fungus, *Beauveria bassiana*

**DOI:** 10.1038/s41598-020-78910-1

**Published:** 2021-01-08

**Authors:** Laila Gasmi, Sehyeon Baek, Jong Cheol Kim, Sihyeon Kim, Mi Rong Lee, So Eun Park, Tae Young Shin, Se Jin Lee, Bruce L. Parker, Jae Su Kim

**Affiliations:** 1 Department of Agricultural Biology, Jeonbuk National University, Jeonju, 54596 Korea; 2 Department of Microbiology and Cell Science, University of Florida, Gainesville, FL 32611-0700 USA; 3 Entomology Research Laboratory, University of Vermont, 661 Spear Street, Burlington, VT 05405-0105 USA; 4 Department of Agricultural Convergence Technology, Jeonbuk National University, Jeonju, 54596 Korea

**Keywords:** Fungi, Microbiology

## Abstract

*Beauveria bassiana* is a species complex whose isolates show considerable natural genetic variability. However, little is known about how this genetic diversity affects the fungus performance. Herein, we characterized the diversity of genes involved in various mechanisms of the infective cycle of 42 isolates that have different growth rates, thermotolerance and virulence. The analysed genes showed general genetic diversity measured as non-synonymous changes (NSC) and copy number variation (CNV), with most of them being subjected to positive episodic diversifying selection. Correlation analyses between NSC or CNV and the isolate virulence, thermotolerance and growth rate revealed that various genes shaped the biological features of the fungus. Lectin-like, mucin signalling, Biotrophy associated and chitinase genes NSCs correlated with the three biological features of *B. bassiana*. In addition, other genes (i.e. *DNA photolyase* and *cyclophilin B*) that had relatively conserved sequences, had variable CNs across the isolates which were correlated with the variability of either virulence or thermotolerance of *B. bassiana* isolates. The data obtained is important for a better understanding of population structure, ecological and potential impact when isolates are used as mycoinsecticides and can justify industrialization of new isolates.

## Introduction

Entomopathogenic fungi (EPF) have evolved as the commonest cause of microbial disease in insects being the most diverse group^1–3^, occurring in wide range of habitats including agricultural and urban settings^4^. They specifically infect arthropods and have minimal impact on natural ecosystems. Despite their diversity, few genera mainly *Beauveria*, *Metarhizium*, *Isaria* (renamed as *Cordyceps*) and *Lecanicillium* (renamed as *Akanthomyces*), have been developed for use against a wide variety of pests^5,6^. *Beauveria bassiana* is one of the most studied species and its isolates have been commercialized worldwide as mycoinsecticides^7,8^. This species is composed of diverse isolates able to exploit a wide range of environments and has a broad range of arthropod hosts^9–11^. Although many *B. bassiana*-based mycoinsecticides have been produced worldwide, they are still underused because of industrialization conflicts concerning how the isolates are different. To avoid such conflicts and justify their different properties, a better evaluation of the genetic diversity and whether this diversity affects the isolate performance is indispensable.

Characterization of *B. bassiana* isolates revealed it is a species complex with several cryptic species showing considerable natural genetic heterogeneity^12–14^. However, how this genetic diversity affects the biological features of the fungus is not well studied. It has been shown that different genetic groups of *B. bassiana* are associated with different habitat types and environment conditions. Nevertheless, there is no or limited evidence that supports the association of genetic diversity and geographic localization, host preference or other phenotypical changes^15,16^. A phylogenetic study of *B. bassiana*^12^ showed it is paraphyletic consisting of two unrelated clades. Devi et al*.* suggested that *B. bassiana* exhibits a predominantly clonal structure, hinting at species divergence which leads to cryptic speciation^17^. In 2010, Ghikas et al*.* analysed *B. bassiana* isolates’ mitochondrial intergenic region, showing the subdivision of the isolates into seven clusters with common climate characteristics^18^. So far, the fungus phylogenetic analyses based on general genetic markers could not definitely clarify the isolate differentiation and did not resolve the origin of their observed variability. Therefore, focusing on the genetic variability of specific functional genes would provide further information on this variability.

In a previous study, we observed sequence variability of a number of *B. bassiana* genes between the isolate, JEF-007 and ARSEF2860^19^. Therefore, we selected genes that might be involved in various mechanisms of the fungal infective cycle and were induced following infection of the bean bug by the isolate JEF-007^19^. We first characterized the genetic diversity of the selected genes in isolates from divergent geographic localizations and assessed the possible selection pressures that have driven such diversity. We thereafter checked the correlation of the genetic diversity with several biological features of the fungal isolates, such as fungal growth, virulence against two insects (the mealworm *Tenebrio molitor* and the silverleaf whitefly *Bemisia tabaci*), and thermotolerance. This knowledge is of great importance for better understanding population structure, gene flow, isolate typing, ecological and potential impact when used as an insect biological control agent^20,21^. In addition, correlating the genetic diversity to functional variations would allow distinguishing new cryptic isolates from the commercialized ones to broaden the available mycoinsecticides and control a wider range of insect pests.

## Results

### Genetic diversity of *B. bassiana* isolates

**Figure 1:**
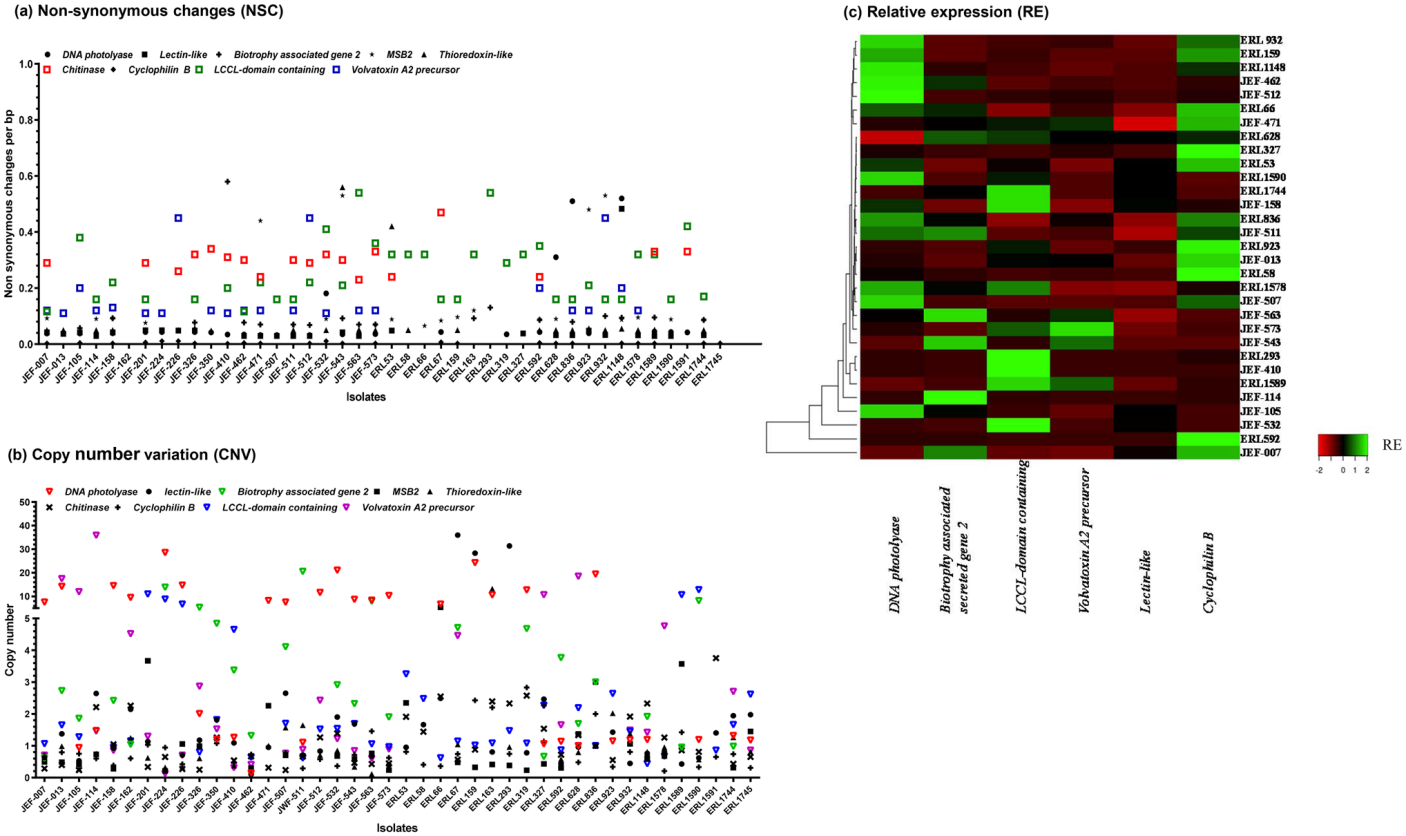
Genetic diversity of *B. bassiana* isolates. The diversity was analysed with non-synonymous change (NSC) (**a**) and copy number variation (CNV) (**b**) of nine genes in 42 *B. bassiana* isolates. The graphs were plotted using GraphPad Prism 5 (Inc., San Diego, CA, USA), and the coloured symbols represent genes having high NSC and CVN, respectively. (**c**) Heatmap representing relative expression of six selected genes in different *B. bassiana* isolates. The heatmap was represented through the online tool (http://www.heatmapper.ca/expression).

Forty-two isolates with divergent geographic distribution were analysed for their internal genetic diversity considering two parameters: the genes sequences variability and the copy number variation (CNV). Each gene sequence from the different isolates were aligned one by one and compared to detect non-synonymous changes (NSC) inside the coding regions, as these substitutions affect the amino acid sequence. High polymorphism was detected for *chitinase*, *volvatoxin A2 (VVA2) precursor* and *LCCL-domain containing *(*LD*) (Fig. 1a). Their corresponding phylogenetic trees showed that the isolates were grouped into three to four different clades (Figs. S1, S2, S3). Low polymorphism was detected for lectin-like, DNA photolyase*,* thioredoxin-like (Trx) and cyclophilin B genes (Fig. 1a, Figs. S1, S2, S3). Comparing the number of sequences that showed high NSC values in each isolate, JEF-410, JEF-471, JEF-532, JEF-543, ERL53, ERL932 and ERL1148 had the highest divergence with at least 3 among the 9 analysed genes showed NSC per bp values ≥ 0.2. The studied genes were as well characterized for their CNV. *Lectin-like*, *signalling mucin* (*MSB2*), *Trx*, *cyclophilin B* and *chitinase* showed low CNV and contained from 1 to 4 copies across all or most of the isolates (Fig. 1b). However, *DNA photolyase*, *VVA2 precursor*, *LD* and *biotrophy associated secreted gene 2 *(*BAS2*) showed high CNV and contained high copy numbers in several isolates (Fig. 1b). The NSC and CNV variability between *B. bassiana* isolates was accompanied with a variability in the selected gene expression pattern (Fig. 1c). Relative expression to the housekeeping gene *gamma actin* of six selected genes (*DNA photolyase*, *VVA2 precursor*, *LD*, *lectin-like*, *BAS2* and *cyclophilin B*) showed variable expression between the different isolates except for *lectin-like* expression which showed similar expression in all the selected isolates (Fig. 1c).

### General positive selection pressures on *B. bassiana* genes

**Figure 2:**
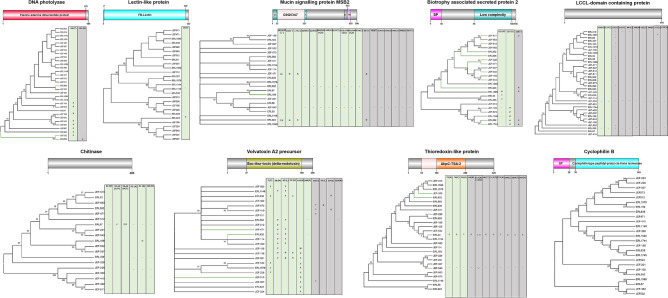
Positive selection pressures in nine genes of *B. bassiana* isolates. In each gene, domain architecture, phylogenetic relationship, and changes of amino acids were analysed. In the phylogenetic trees, the isolates with green branches are under positive diversifying selection.

We used aBSREL analyses to check whether *B. bassiana* isolates are subjected to diversifying positive selection pressures which lead to increased genetic variability. All the genes, except *lectin-like* and *cyclophilin B*, were found to be subjected to episodic diversifying selection (Fig. 2, Table S2). Compared to the other isolates, nine isolates received strong diversifying selection pressure. Four genes were subjected to diversifying pressures in the case of JEF-532; three genes in the case of ERL836 and ERL932 and two genes in the case of JEF-201, JEF-350, JEF-410, JEF-471, JEF-511 and JEF-543 (Table S3). To further characterize the selection pressures exerted on the various genes, we assessed positive and negative selection pressures (leading to increased genetic diversity or purifying the population from acquired mutations, respectively) in individual codons combining FUBAR, MEME and SLAC analyses. The most affected genes with selection pressures on individual codons were *LD* (18 codons corresponding to 10.23% of the total sequence), *Trx* (18 codons corresponding to 12.08% of the total sequence) and *VVA2 precursor* (27 codons corresponding to 12.62% of the total sequence) (Table S2). To locate the sequence variation regions of the genes, the analytical results were combined with alignment data and the final codon changes were represented in Fig. 2. In general, all or most of these codons are included in the conserved domain or specific sites detected in each gene sequence (Fig. 2).

### Diverse biological properties of *B. bassiana* isolates

**Figure 3:**
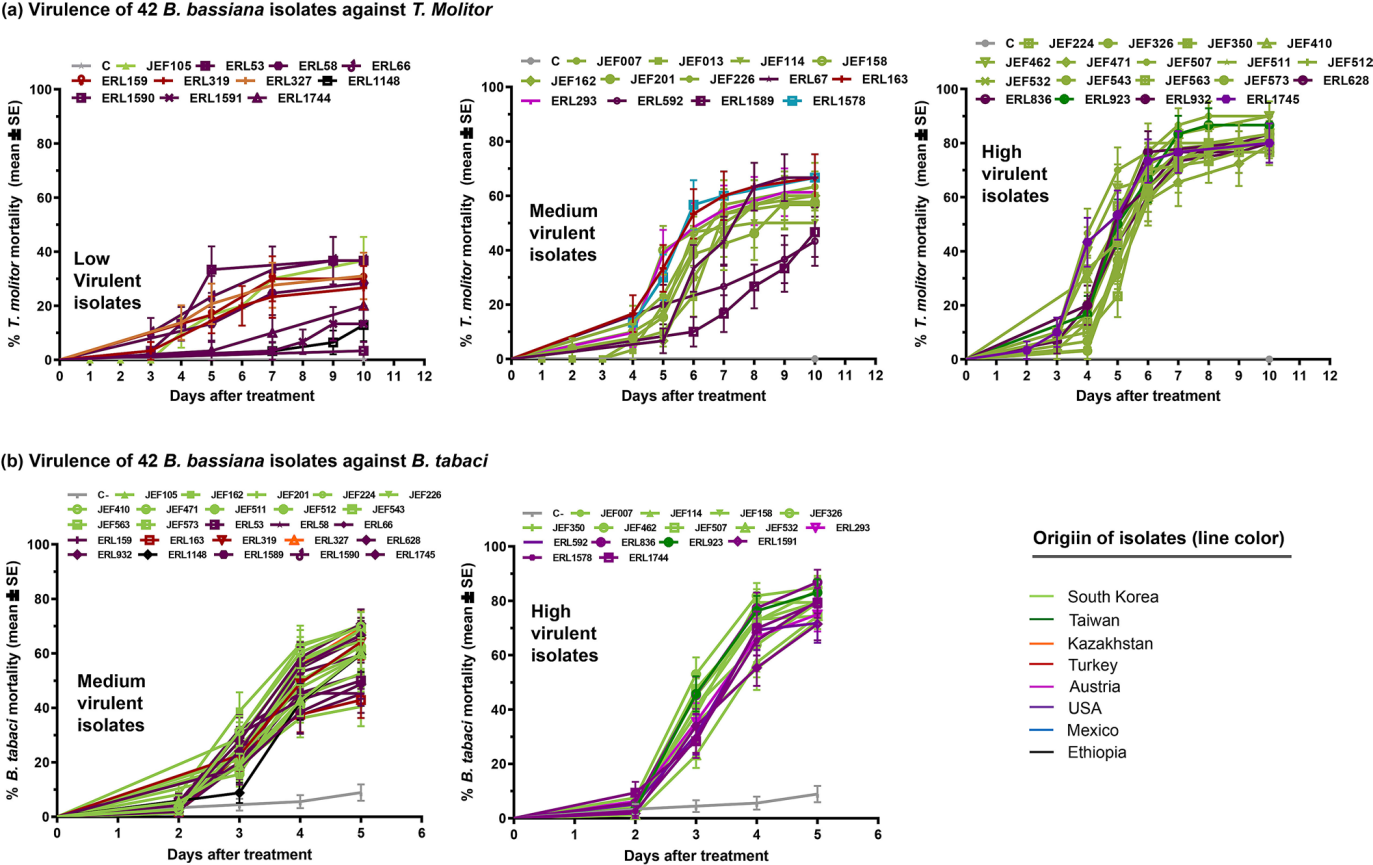
Virulence of 42 *B. bassiana* isolates against *T. molitor* (**a**) and *B. tabaci* (**b**)*.* Virulence against larvae of the two species is represented as cumulated mortality per day. Isolates killing between 0 and 40% are considered low infective, those killing between 41 and 70% are medium infective isolates and high infective isolates are those killing between 71 and 100%. The mortality curves were plotted using GraphPad Prism 5 (Inc., San Diego, CA, USA).

The 42 isolates were tested for their virulence against two insect species, the mealworm *T. molitor* (Coleoptera: Tenebrionidae) and the whitefly *B. tabaci* (Hemiptera: Aleyrodidae). The virulence of the fungus against *T. molitor* showed high variability among the different isolates, with the Korean isolates showing general high virulence with 13 among 21 isolates were highly virulent (JEF-224, JEF-326, JEF-350, JEF-410, JEF-462, JEF-471, JEF-507, JEF-511, JEF-512, JEF-532, JEF-543, JEF-563 and JEF-573) and only 1 showed low virulence (JEF-105) (Fig. 3a). Among the other isolates, only ERL923 collected in Taiwan and the isolates ERL-628, ERL-836, ERL-932 and ERL1745 collected in USA showed high virulence (F_41,1275_ = 17.49; *p* < 0.0001) (Fig. 3a). Among the high virulent isolates, the isolates JEF-410, JEF-462 and ERL923 caused higher mortality than other isolates (86.7 ± 6.2%, 90 ± 5.5% and 86.7 ± 6.2%, 10 days after treatment (dat), respectively). In counterpart 11 isolates (JEF-105, ERL53, ERL58, ERL66, ERL159, ERL319, ERL327, ERL1148, ERL1590, ERL1591 and ERL1744) were not virulent or showed low virulence against *T. molitor*. Among them, ERL1148, ERL1590 and ERL1591 killed < 20% of the treated larvae 10 dat (12.9 ± 6.0%, 3.3 ± 3.3% and 13.3 ± 6.2%, respectively) (Fig. 3a, Table S4).

In difference to the high variability of the isolate virulence against *T. molitor*, each of the 42 isolates showed high to medium virulence against *B. tabaci*. All the isolates killed > 40% of the treated larvae 5 dat (F_41,708_ = 2.308; *p* < 0.0001) (Fig. 3b). The highest mortality percentages were detected for the isolates JEF-350, JEF-462, ERL836 and ERL932 (83.9 ± 4.7%, 84.9 ± 4.4%, 86.8 ± 4.6% and 83.1 ± 4.9%, respectively 5 dat), meanwhile the least virulent isolates against *B. tabaci* were JEF-226, ERL66, ERL159 and ERL163 (40.4 ± 7.2%, 45.1 ± 7%, 45.3 ± 5.8% and 42.9 ± 6.6%, respectively 5 dat) (Fig. 3b, Table S4).

**Figure 4:**
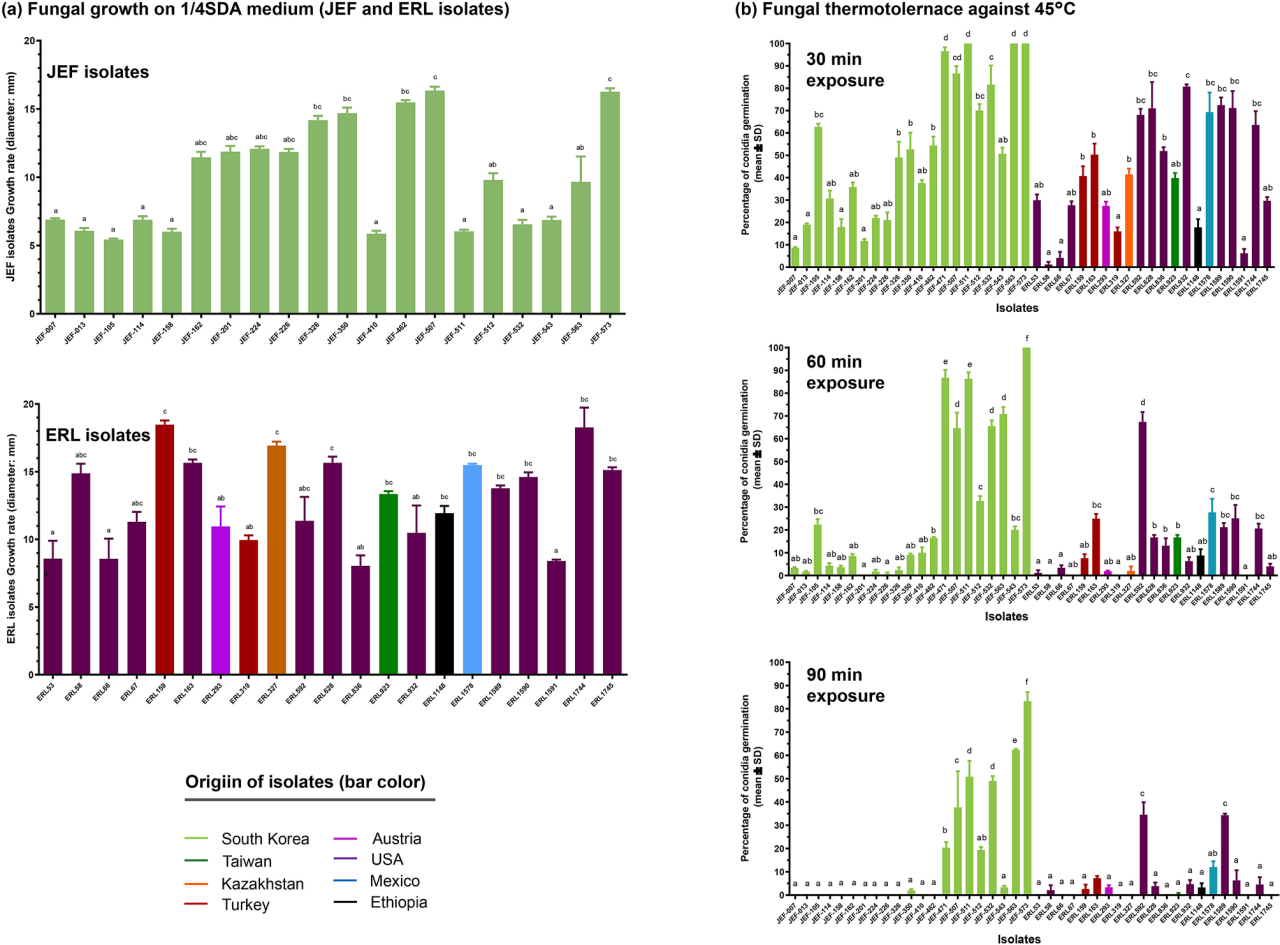
Fungal growth and thermotolerance. Fungal growth rate was represented as the diameter of colonies cultured on ¼ SDA medium at 25 °C for 7 days (**a**), and the thermotolerance was represented as conidial germination rate after 30, 60, and 90 min of heat exposure to 45 °C (**b**). In (**a**), growth rate of JEF and ERL isolates were represented separately to better visualize the graphs. In (**a**) and (**b**) graphs, small case letters denote statistical differences. The graphs were plotted using GraphPad Prism 5 (Inc., San Diego, CA, USA).

In addition to the fungal virulence, growth rate and stress resistance are important factors that are considered in developing fungi-based insecticides. Therefore, we characterized the growth rate and thermotolerance of the 42 isolates. Variability in the growth rate measured as diameter of the fungal colonies was detected (Fig. 4a). The lowest growth was observed for 9 isolates collected in Korea (JEF-007, JEF-013, JEF-105, JEF-114, JEF-158, JEF-410, JEF-511, JEF532 and JEF-543), in addition to 3 isolates collected in the USA (ERL53, ERL66 and ERL1591) (Fig. 4a). The highest growth rate was detected for the isolates JEF-573, ERL159, ERL327 and ERL628 collected in Korea, Turkey, Kazakhstan, and USA, respectively (F_37,142_ = 13.1, *p* < 0.0001) (Fig. 4a).

We assessed the thermotolerance of the studied isolates by checking the conidial germination rate after 30, 60 and 90 min of exposure to 45 °C heat (Fig. 4b). After 30 min of heat, conidial germination rate of JEF-007, JEF-013, JEF-158, JEF-201, ERL58, ERL66, ERL319, ERL1148 and ERL1591 drastically decreased to lower than 20% (Fig. 4b) (F_38,77_ = 52.94, *p* < 0.0001). The isolates JEF-511, JEF-532, JEF-563 and JEF-573 showed high thermal tolerance with germination of 50.79 ± 11.88%, 49.03 ± 3.59%, 62.39 ± 0.62% and 83.3 ± 6.68%, respectively, of the observed conidia, after 90 min exposure to 45 °C (F_38,77_ = 66.19, *p* < 0.0001) (Fig. 4b).

### Correlation of selected genes sequence variability with *B. bassiana* biological features

**Figure 5:**
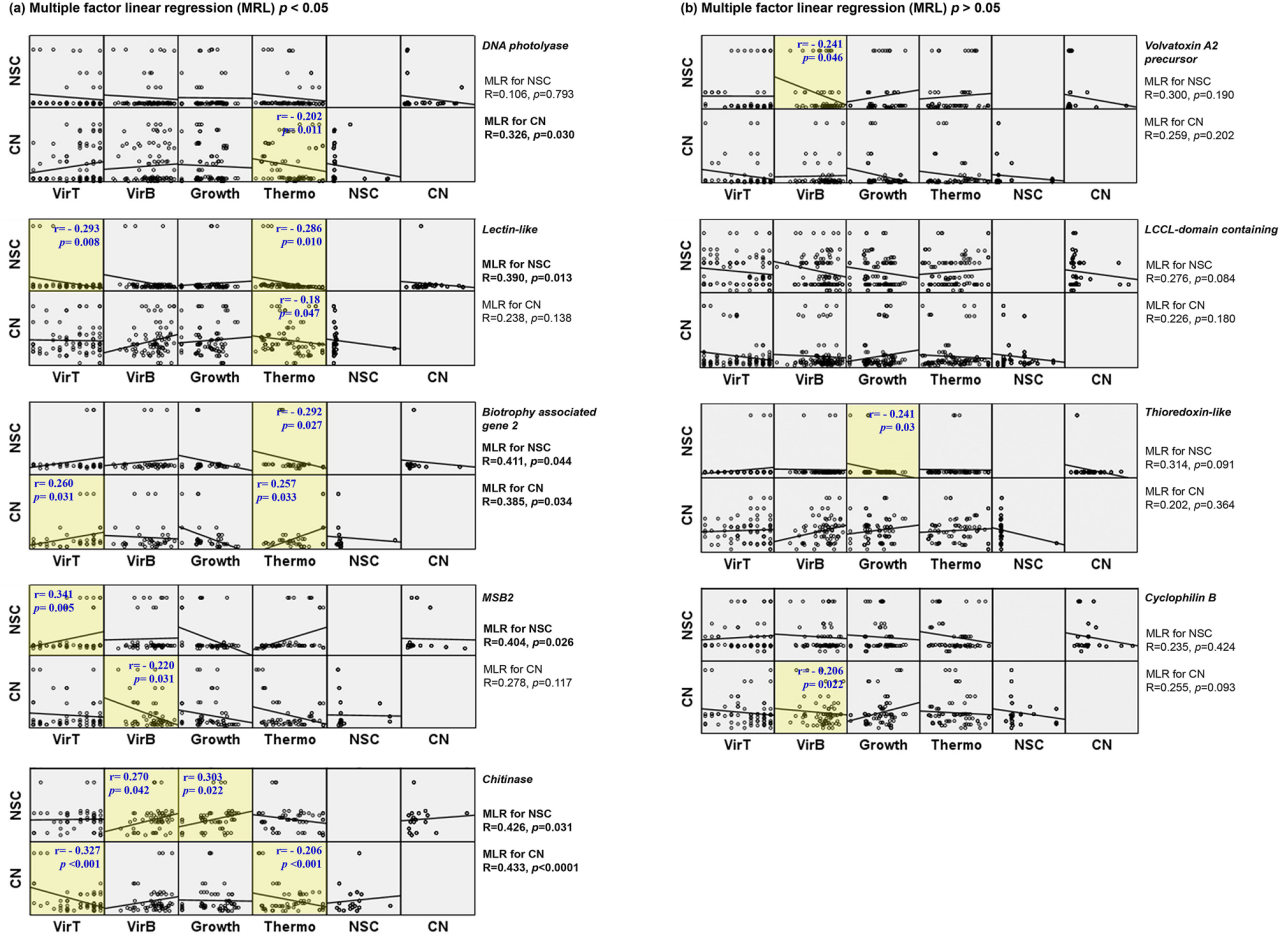
Multiple factor linear regression (MRL) model to describe the correlation between four fungal phenotypic variables [Virulence against *T. molitor* (VirT) and *B. tabaci* (VirB), fungal growth (Growth) and thermotolerance (Thermo)] and genotypic dependents (NSC (nonsynonymous change) or CN (copy number)). Genes were divided into significant (*p* < 0.05) MRL group (**a**) and non-significant (*p* > 0.05) MRL group (**b**). In each gene, regression coefficient and *p* value of MRL was provided at the right of the multi scatter plot. Additionally, simple linear regression was conducted between each individual variable and dependent, and significant simple regression (*p* < 0.05) was marked with yellow background. Pearson r and p values were represented for each of the statistically significant simple linear regression. The different graphs were plotted using SPSS program ver. 19.0 (SPSS, Inc, Chicago, IL, USA).

To check how the genes sequences variability represented as NSC affect the isolate biological properties, multiple factor linear regression was performed between each gene NSC per bp and the different studied biological features (virulence against *T. molitor*, virulence against *B. tabaci*, growth rate and thermotolerance). The obtained results showed that NSCs of *Lectin-like gene* (R = 0.39, *p* = 0.013), *BAS2* (R = 0.411, *p* = 0.044), *MSB2* (R = 0.404, *p* = 0.026) and *chitinase* (R = 0.426, *p* = 0.031) are correlated with the studied biological features (Fig. 5a), suggesting these genes are involved in several phenotypic variables of the fungus.

In addition, simple linear regression and Pearson correlation analyses have been performed. NCSs of *Lectin-like* showed weak negative correlations (− 0.3 < r) (r = − 0.293, *p* = 0.008) with *T. molitor* lavae mortality at the end of the bioassay (10 dat). Meanwhile, *MSB2* NSCs showed moderate positive correlation (0.3 < r < 0.5) with the mortality (r = 0.341, *p* = 0.005) (Fig. 5; Table S5). In the case of virulence against *B. tabaci*, mortality at the end of the assay (5 dat) was correlated with the NSCs of *chitinase* and *VVA2 precursor* (r = 0.27, *p* = 0.042 and r = −0.241, *p* = 0.046, respectively). *Chitinase* NSC was as well positively correlated with the isolates growth measured as the diameter of the fungus colony grown for 7 days (r = 0.303, *p* = 0.022), meanwhile a negative correlation was observed for *thioredoxin-like gene* NSC (r = −0.241, *p* = 0.03). Finally, *lectin-like* and *BAS2* genes NSCs were negatively correlated with the isolates thermotolerance (r = −0.286, *p* = 0.01 and r = −0.292, *p* = 0.027, respectively) (Fig. 5).

### Correlation of selected genes copy number variation with *B. bassiana* biological features

In addition to sequence variability, copy number variation can be an extra source of functional variability of genes. For this reason, we checked how CNV would affect the fungus performance. Multiple factor linear regression was performed between each gene CNV and the four studied biological features (virulence against *T. molitor*, virulence against *B. tabaci*, growth rate and thermotolerance). Results showed that CNV of *DNA photolyase* (R = 0.326, *p* = 0.03), *BAS2* (R = 0.385, *p* = 0.034), and *chitinase* (R = 0.433, *p* < 0.0001) are correlated with the biological features (Fig. 5a), suggesting copy number variability of these genes might affect several phenotypic variables of the fungus.

In addition, simple linear regression and Pearson correlation analyses have been performed. CNV of *chitinase* showed moderate negative correlation (r = −0.327, *p* < 0.0001) with *T. molitor* larvae mortality (10 dat). Meanwhile, *BAS2* CNV was positively correlated with *T. molitor* mortality (r = 0.26, *p* = 0.031) (Fig. 5, Table S5). In the case of virulence against *B. tabaci*, mortality at the end of the assay was negatively correlated with the CNV of *MSB2* and *Cyclophilin B* (r =−0.22, *p* = 0.031 and r =− 0.206, *p* = 0.022, respectively). Finally, four genes CNV were correlated with the isolates thermotolerance. *BAS2* CNV was positively correlated with the thermotolerance (r = 0.257, *p* = 0.033), meanwhile *DNA photolyase*, *lectin-like* and *chitinase* genes CNV showed weak negative correlations (r = −0.202, *p* = 0.011, r = −0.18, *p* = 0.047 and r = −0.206, *p* < 0.0001, respectively) (Fig. 5, Table S5).

## Discussion

*B. bassiana* has long been recognized as an heterogeneous group of isolates^22^, however evidences that correlate their genetic variability with the fungal performance are very limited. Herein, we analysed the sequence variability of nine genes in 42 isolates, considering NSC per bp. The obtained results revealed high general sequence variability, except for *DNA photolyase* and *Cyclophilin B*. In addition, several genes showed high CNV among the different isolates, particularly, *DNA photolyase*, *BAS2*, *LD* and *VVA2 precursor*. Interestingly, the latter two genes had high NSC and high CNV, suggesting the multiple copy numbers could have resulted in the sequence variability among the different isolates. CNV provides backgrounds for gene family expansion and diversification, which is an important evolutionary force^23^.

Out of the main factors changing the level of genetic variability, selection pressures might drastically reduce the variability among populations, or might increase amino acid diversity at various phylogenetic levels promoting adaptation and variability^24^. Studying such diversification is useful to identify adaptive radiation and gain information about the origin of diversity. Indeed, it has been established that since pathogens tend to have shorter generation times than their hosts, their capacity to generate new virulence factors through random mutations and selection is relatively high^25^. Among the studied genes, seven were found to undergo positive diversifying selection pressures that might either enforce the genetic functions or lead to differentiation towards new adaptive functions. In depth analyses revealed codons under negative selection pressures, which in general purge changes that cause deleterious impacts on the recipient organism’s fitness, suggesting functional adaptations of the proteins are limiting the fixation of new mutations that would negatively affect *B. bassiana*. These results altogether point towards transient positive selection followed by purifying selection to select and stabilize functional adaptations.

In addition to sequence variability of genes among *B. bassiana* isolates, they showed different performances. Multiple factor linear regression analyses showed statistically significant correlation between *BAS2* and *chitinase* NSC and CNV with the fungus virulence, growth rate and thermotolerance indicating these genes variability would be important factors in determining the fungus performance. In addition, *MSB2* and *lectin-like* NSCs and *DNA photolyase* CNV were as well correlated with the fungus studied functional properties. These results suggest that the observed variability in virulence, growth and thermotolerance of *B. bassiana* isolates is dependent on genes sequence and copy number diversity.

Further correlation analyses revealed *Lectin-like*, *MSB2, chitinase* and *VVA2 precursor* NSCs were correlated with the fungal virulence against *T. molitor* or *B. tabaci* larvae. *MSB2* and *chitinase* increased NSCs were positively correlated with the mortality suggesting an increased diversity improve the fungus virulence. In counterpart, *lectin-like* and *VVA precursor* NSCs were negatively correlated with the virulence suggesting conserved functional proteins in the high virulent isolates. While lectin, MSB2 and chitinase proteins have been described to be involved in fungal virulence and stress response^26–29^, VVA2 precursor function was not studied in EPF. VVA2 precursor belongs to the delta-endotoxin family of proteins. The delta-endotoxin domain is responsible for direct toxicity to insect hosts in entomopathogenic bacteria^30^. Similarly, VVA2 isolated from the mushroom, *Volvariela volvacea* is a pore-forming protein that disrupts target cell membranes and has haemolytic and cytotoxic activities^31,32^. Similarly, we suggest this protein in *B. bassiana* follows the same mode of action described for delta endotoxins.

Due to the complex life history of *B. bassiana*, many if not most of the virulence genes studied so far are involved in several steps of the infection cycle^33^. A well-studied example is MSB protein. In yeast and several phytopathogenic fungi, MSB2 is involved in surface recognition and MAP kinase activation^28^. MSB2 of *B. cinerea* functions as surface sensor of germlings and hyphae that triggers infection structure formation^28^. In *Candida albicans,* a common member of human gut flora, MSB2 regulates the fungal thermal stress and is required for survival and hyphae formation at high temperature^27^. Accordingly, our data suggest that *B. bassiana* MSB2 protein might be involved in the regulation of the fungus stress response and virulence. However, our data suggest that *VVA2 precursor* is not involved in thermotolerance, nor growth rate, but is directly involved in toxicity of the fungus. Further studies are needed to confirm the suggested function of this gene and its mode of action.

Moreover, correlation analyses revealed *lectin-like* and *BAS2* sequences variability were negatively correlated with the fungal thermotolerance. Recently, BAS proteins are being characterized in phytopathogenic fungi. A BAS protein from Gray mold-causing *Botryis cinerea* enhances fungal tolerance to abiotic stressors^34^. Another BAS protein from anthracnose-causing *Colletotrichum gloeosporioides* was shown to be involved in the fungal asexual reproduction and penetration of host tissue where the protein is required for the secretion of proteins important for fungal pathogenicity^35^. Accordingly, our BAS2 protein would be directly involved in tolerance to abiotic stress and might be required for the fungal infection establishment. Finally, *chitinase* and *thioredoxin-like* genes NSCs were differentially correlated with the isolates growth rate. Indeed, *B. bassiana* Trx mutants exhibited changes in the antioxidant response, conidiation, germination, thermotolerance, UV-B resistance, and virulence, confirming this protein family is indispensable for the fungal growth among other biological features^36^.

As another factor driving genetic diversity, we analysed correlations between CNV and the biological performance of the fungus. CNV is being recognized as important in determining the differences between individual humans as SNP, and appears to be a major driving force in evolution^37^. Correlation analyses showed that CNV data could be a separate parameter of genetic diversity driving phenotypic variability in *B. bassiana*. *DNA photolyase* and *cyclophilin B,* having low NSCs per base pair, had variable CNV which correlated with the tested fungal biological features. *DNA photolyase,* which plays vital roles in conidiogenesis, stress response and development^38,39^, CNV was correlated with the fungus thermotolerance. Meanwhile, *cyclophilin B* CNV was correlated with virulence against *B. tabaci*. In addition, *BAS2*, *chitinase*, *MSB2* and *lectin-like* genes, which NSCs correlated with at least one of the fungal biological features, showed variable CNs that correlated with the isolates’ virulence and/or thermotolerance.

CNVs can affect the level of gene expression indirectly through modification of the chromatin environment or directly by altering the gene dosage. Such alteration would generate phenotypic changes by dramatic over- or under-expression of specific gene(s). Indeed, several genes showed variable constitutive expression among *B. bassiana* isolates. Such expression variability of several proteins can affect basic cellular functions^40–42^, however further studies are required to correlate the CNV with the genes expression patterns and their functional changes. The three genes which their CNV correlated with the fungus biological features, showed negative correlation where the lowest performance corresponded to high CVN. Changes in DNA copy number have been identified as deleterious to the recipient hosts being associated with diseases and developmental abnormalities^40^. However, in some cases they were described as a source of adaptive potential conferring adaptive advantages to the corresponding organisms^40,43,44^. In fungi, increasing reports are suggesting that CNV is an important, underestimated, factor of genome biology and evolution^45^, and that in several cases CNV is independent of SNP variation^43^. More recently, it has been described that ~ 10% of *Aspergillus fumigatus* genome is CN variable increasing the fungal genetic variability^46^. The fungal gene CNV has been associated with phenotypic variation and adaptation^43,46–48^. These observations are in accordance with our data in which genes with conserved sequences have high variability in CN and vice versa.

Herein, we aimed to get an insight on the genetic diversity of specific genes and the effect of such variability on the fungal performance. We detected general high variability of the gene sequences and gene CN, and general positive diversifying selection pressures suggesting *B. bassiana* is evolving towards more diversified isolates. This variability confirms the heterogeneity of *B. bassiana* isolates and support the selection of different isolates to develop more effective fungal biopesticides. We further suggest that the gene variability is shaping the biological features of the fungus since they were reflected in variability of the growth rate, virulence and thermotolerance. The accumulated data in this work can be used as a starting point to select highly virulent isolates, to distinguish the different isolates from the same species and to characterize the function of genes directly implicated in the toxicity of the fungus towards the insect host. In addition, the characterization of genetic variability among *B. bassiana* isolates provide essential biological information required for biopesticide registration and allow the monitoring of the biological characteristics of the biopesticide.

## Materials and methods

### Fungal isolates

Twenty-one *B. bassiana* isolates were collected from soil samples in South Korea and maintained in the Jeonbuk (old name: Chonbuk) National University Collection of EPF, Korea (JEF-007, JEF-013, JEF-105, JEF-114, JEF-158, JEF-162, JEF-201, JEF-224, JEF-226, JEF-326, JEF-350, JEF-410, JEF-462, JEF-471, JEF-507, JEF-511, JEF-512, JEF-532, JEF-543, JEF-563 and JEF-573). Other 21 isolates were provided by the Entomology Research Laboratory, University of Vermont, USA (ERL53, ERL58, ERL66, ERL67, ERL159, ERL163, ERL293, ERL319, ERL327, ERL592, ERL628, ERL836, ERL923, ERL932, ERL1148, ERL1578, ERL1589, ERL1590, ERL1591, ERL1744 and ERL1745) and stocked in glycerol at -80 °C. These isolates were isolated from different regions in the world. An isolate was collected from Taiwan and one collected in Kazakhstan. Fourteen originated from the American continent being one from Mexico and the rest from different parts of the USA. Finally, four were collected in Europe (3 in Turkey and 1 in Austria) and one isolate was collected in Ethiopia as representative of the African continent.

The 42 isolates were cultured and maintained on one-quarter strength Sabouraud dextrose agar (1/4 SDA; Difco, Lawrence, KS, USA) in darkness at 25 ± 1 °C, and most of them had different virulence against *T. molitor* in a preliminary screening.

### Insects

A colony of mealworm, *T. molitor* was obtained from the Rural Development Administration, Korea (RDA; http://www.rda.go.kr/foreign/ten/index.jsp) and reared in plastic cages (20 × 20 × 10 cm^3^) with mesh for ventilation. Wheat bran and a piece (ca. 3 × 3 cm^2^) of Chinese cabbage per cage were fed to *T. molitor* larvae and adults*.* A colony of whitefly, *B. tabaci* was obtained from the RDA and reared in greenhouse in Jeonbuk National University. Based on the results of a host plant preference test comparing eggplants and tomatoes, eggplants (Asia Seed Co., Korea) were selected as the primary food source. To perform the virulence assays, mated whitefly adults were collected and placed on tomato plants (Danong Co., Korea) in a secured container for one day, then laid eggs were separated from adults and transferred on fresh leaves to a new cage (35 × 35 × 40 cm^3^). All breeding boxes were placed at 25 ± 2 °C, 40 ± 5% of relative humidity with a 16:8 (L/D) photoperiod.

### DNA extraction, gene amplification and sequence analyses

From our previous RNA-seq of infecting *B. bassiana* isolate, *DNA photolyase*, *Trx* and *MSB2* (stress protection), *Chitinase* (enzymatic activity), *BAS2* and *Cyclophilin B* (fungal development and conidiogenesis), *Lectin-like protein* and *LD containing domain protein* (host immunity and development arrest), and *VVA2 precursor* (toxicity) were selected for this study. The genomic DNA (gDNA) was extracted from each isolate using CTAB extraction buffer (Biosesang, Gyeonggi-do, Republic of Korea) as follow. Five hundred microliters of CTAB were heated at 65 °C for 15 min then beta-mercaptoethanol was added (1:100 (v/v)). Fungal hypha were ground into the prepared solution, vortexed and incubated at 65 °C for 30 min. Afterwards, 400 µl of chloroform: isoamyl alcohol (24:1 (v/v)) was added to the solution, mixed and centrifuged at 12,000*g* for 10 min at 4 °C. The aqueous phase was transferred into a new tube containing 400 µl of isopropanol, then incubated at room temperature for 5 min. Following centrifugation (12,000*g* for 5 min, 4 °C), the supernatant was removed, and the pellet dried using a speed vacuum for 5 min at 30 °C. The pellet was then solubilized in 600 µl 1 × TE buffer and 300 µl of phenol: chloroform: isoamyl alcohol (25:24:1 (v/v/v)) was added and the solution was centrifuged (12,000*g* for 5 min, 4 °C). The aqueous phase was transferred to a new tube and the RNA was degraded using 1 µl of RNase (10 µg/ml). After centrifugation, the pellet was washed with 70% ethanol then dried to finally solubilize it in DNase free water.

The gDNA was used to amplify the selected genes using specific primers (Table S1) designed from each gene contig obtained from transcriptome of the isolate JEF-007 (NCBI accession number: PRJNA309019). PCR products of each gene were sequenced (Macrogen, Soul, Republic of Korea) then open reading frame (ORF) and protein sequences were identified using SeqMan and EditSeq programs (DNA star package, Madison, W). The predicted ORFs and amino acid sequences were aligned using the ClustalX software^49^ and visualized in the GenDoc program^50^. Evolutionary distance was calculated for aligned amino acid sequences by Maximum-likelihood method and the phylogenetic trees were conducted with MEGA7^51^. Reliability of an inferred tree was determined using the bootstrap test (1000 replicates) and the bootstrap values were reported over 100 for a clearer view of the phylogenetic tree branches. Conserved domains and transmembrane regions were identified using SMART (http://smart.embl-heidelberg.de/smart/set_mode.cgi) and domain architectures were plotted using DOG 2.0 (http://dog.biocuckoo.org/).

The gDNA was used to determine the CN of gene using the 2^ΔΔCt^ method^52,53^. Specific primers were designed (Table S1) and qPCR was performed using Thunderbird Syber qPCR Mix (QPS-201, TOYOBO, Japan) in 96-well Bio-Rad CFX96 Real-Time PCR System (Bio-Rad, USA). *Gamma-actin* (NCBI accession number: HQ232398.1) was used as an internal control to normalize the obtained Cts. The experiment was conducted thrice. The copy numbers of the different genes were determined bioinformatically from a whole genome sequencing of the isolate ERL836 and those values were used as control to assess the CN of each gene in the rest of isolates.

### RNA extraction

Total RNA extraction was performed from 31 isolates using Trizol (Invitrogen) according to the manufacturer protocol. Briefly, each isolate was incubated in ¼ SD medium at 25 °C with shaking at 100 rpm for 72 h. The cultures were centrifuged, and the collected pellet was grinded in 300 µl Trizol. Following 5 min incubation at room temperature (RT), 0.2 ml chloroform was added. After 5 min incubation at RT, the tubes were centrifuged for 15 min at 12,000*g* and 4 °C. The aqueous phase was transferred into new tubes and equal volume of isopropanol was added, and the tubes were incubated for 10 min at 4 °C. After centrifuging in the same conditions described above for 10 min, the pellet was washed with 1 ml 75% ethanol, dried and solubilized in 50 µl nuclease free water. 0.5 µg total RNAs were subjected to DNase treatment and were used to synthesize cDNAs following the protocol of AccuPower RocketScript RT PreMix (Bioneer). Specific primers were designed (Table S1) and qPCR was performed using Thunderbird Syber qPCR Mix (QPS-201, TOYOBO, Japan) in 96-well Bio-Rad CFX96 Real-Time PCR System (Bio-Rad, USA) to determine the relative expression of the selected genes in the different isolates. *Gamma-actin* (NCBI accession number: HQ232398.1) was used as an internal control and the genes expression was represented as relative expression (RE) to the *Gamma-actin* gene expression using the following formula:

$$ {\text{RE}} = 2^{{ - {\text{Ct}}\,{{gene}}}} {/}2^{{ - {\text{Ct}}\,{{gamma{\text{-}}actin}}}} . $$

Two independent biological replicates were performed and the obtained results were represented in a heatmap using the online tool (http://www.heatmapper.ca/expression).

### Genetic diversity and detection of positive selections

Sequence variability was measured using NSC as those substitutions would affect the protein sequence and function. NSCs were determined using the online Synonymous/Non-synonymous changes analyses program SNAP v2.1.1 (https://www.hiv.lanl.gov)^54^. Values were normalized as NSC per base by dividing the corresponding NS substitution value of each sequence by the sequence ORF size. To determine how evolutionary forces have shaped the fungal genetic diversity, we used the improved statistical method of web server Datamonkey 2.0 (http://www.datamonkey.org/)^55^. Diversifying positive selection pressures are most likely to drive phenotype changes and trigger increased genetic variance. Therefore, we looked for evidences of episodic diversifying selection pressures using the adaptive Branch-Site Effects Likelihood method (aBSREL). This analyses applies models to different branches in the phylogeny that quantify the strength and type of neutral selection estimating the ratio of non-synonymous (d_NS_) to synonymous (d_S_) substitutions^56^. To further evaluate the selection pressures on the fungal genes, three methods computing d_NS_ and d_S_ changes at each codon position were used: FUBAR, MEME and SLAC. FUBAR (Fast Unconstrained Bayesian AppRoximation for inferred selection) uses a Bayesian approach to infer d_NS_ and d_S_ rates on a per-site basis for a given coding alignment assuming that the selection pressure for each site is constant along the entire phylogeny^57^. SLAC (Single Likelihood Ancestor Counting) uses a combination of maximum likelihood and counting approaches to infer d_NS_ and d_S_ rates employing a modified version of the Suzuki–Gojobori counting method^58^. MEME (Mixed Effects Model of Evolution) employs a mixed-effects maximum likelihood approach to test the hypothesis that individual sites have been subjected to episodic positive selection using a genetic algorithm to search multiple-sequence for putative recombination break points, quantifies the level of support for their locations and identifies sequences or clades involved in putative recombination events^59^. Results from the three analyses were compared and checked in the amino acid sequences, then the robust selection events were chosen and represented as eventual codons subjected to the different selection pressure events.

### In vitro fungal virulence assay against insects

The virulence of the isolates was assessed against *T. molitor* and *B. tabaci*. For each isolate, ten 4th instar *T. molitor* were sprayed with 1 ml of 10^7^ conidia/ml of 0.03% siloxane solution, then placed into a Petri dish in which a Whatman membrane paper was laid and humidified with 200 μl distilled water. All plates were maintained at 25 ± 1 C and 40 ± 10% relative humidity in 16:8 (L: D) conditions. Mortality was recorded daily for 10 days. For *B. tabaci*, tomato leaves were infested with about 15 s instar larvae and dipped for 10 s in 10^7^ conidia/ml of 0.03% siloxane solution in each isolate. The infested leaf surfaces were dried at RT for 25 min and placed in a fresh Petri dish with moisturized filter paper. The Petri dishes were kept at 25 ± 1 °C and 40 ± 10% relative humidity conditions. Mortality was recorded daily for 5 days. In both experiments, 0.03% siloxane solution was used as a negative control. Three biological replicates were performed for each of the described assays. Following both virulence assays, correlations between the final mortality (10 dat for *T. molitor* and 5 dat for *B. tabaci* which correspond to the time points where the high virulent isolates killed 100% of the treated larvae) and the genetic diversity of each gene represented as NSC per bp and CNV were assessed.

### Growth rate of the selected isolates

To check the growth rate of the various isolates, 10 µl of 10^7^ conidia/ml were dropped on ¼ SDA medium, then homogenously distributed in an incubator maintained at 25 ± 1 °C. The diameter of the fungal colony was measured daily for 7 days. Three biological replicates were performed. The measured diameters at day 7 representing the fungal growth rate were used to determine the correlations between the growth rate and the genetic diversity of each gene represented as NSC per bp and CNV.

### Thermotolerance assay

The fungal conidia from each isolate were released to a solution of 0.03% siloxane by vertexing for 30 s, and counted using a haemocytometer and then diluted to 1 × 10^7^ conidia/ml. The conidial solutions (1000 µl) in Eppendorf tubes were placed in an incubator adjusted to 45 ± 1 °C for 30, 60, and 90 min. Control conidial suspensions were kept at RT. After incubation, 2 µl of the suspension from each sample was inoculated on ¼ SDA medium and incubated at 25 ± 1 °C for 18 h. The germination rate was microscopically determined by randomly counting the number of germinated and un-germinated conidia among 100 counts (400 ×). Three biological replicates were performed for each of the described assays. The conidial germination rates following incubation at 45 °C for 30 min showed the high variability among the tested isolates. Therefore, we used the obtained results at this time point to determine the correlations between the thermotolerance and the genetic diversity of each gene.

### Data analyses

Data on the numbers of conidia, the growth rate, the percentages of germination rate, and the percentages of dead insect were arc-sine transformed to make all the data set normally distributed, and analysed using a generalized linear model (GLM). The overall analyses were followed by Tukey’s honestly significant difference (HSD) for multiple comparisons. The multiple factor linear regression analyses were conducted to examine the correlation of NSC or CNV with four biological factors (Virulence against *T. molitor* and *B. tabaci*, fungal growth and thermotolerance) under an enter-based prediction option and a Durbin–Watson residual analysis. Additionally, simple linear regressions of the individual two factors were conducted using a Pearson correlation. All the analyses were conducted using a SPSS program ver. 19.0 (SPSS, Inc., Chicago, IL, USA) at the 0.05 (α) level of significance.

## Supplementary Information

Supplementary Informations.

## Data Availability

All sequences access numbers are available in NCBI database. The manuscript is accompanied with supplementary data.
