# Supplementary material for: Gene diversity explains variation in biological features of insect killing fungus, Beauveria bassiana
Source: Sci Rep. 2021 Jan 8;11:91. doi: 10.1038/s41598-020-78910-1 (PMC7794557; doi:10.1038/s41598-020-78910-1)
Supplement: Supplementary file 1 — Supplementary Informations. [file 41598_2020_78910_MOESM1_ESM.pdf]

Supplementary Materials for:

Gene diversity explains variation in biological features of insect killing fungus, *Beauveria bassiana*

Laila Gasmi[a], Sehyeon Baek [a], Jong Cheol Kim [a], Sihyeon Kim [a], Mi Rong Lee [a], So Eun Park [a], Tae Young Shin [a], Se Jin Lee[b], Bruce L. Parker[c], Jae Su Kim [a,d] *

[a] *Department of Agricultural Biology, Jeonbuk National University, Jeonju 54596, Korea*

[b] *Department of Microbiology and Cell Science, University of Florida, Gainesville FL 32611-0700, USA*

[c] *Entomology Research Laboratory, University of Vermont, 661 Spear Street, Burlington, Vermont 05405-0105, USA*

[d] *Department of Agricultural Convergence Technology, Jeonbuk National University, Jeonju 54596, Korea*

*** Corresponding author:**

Jae Su Kim

Email: jskim10@jbnu.ac.kr

DNA photolyase

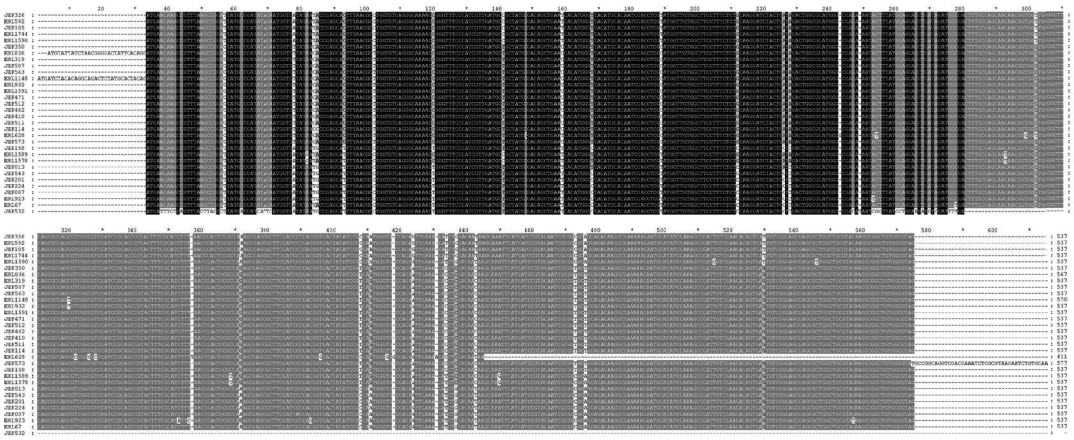

MSB2

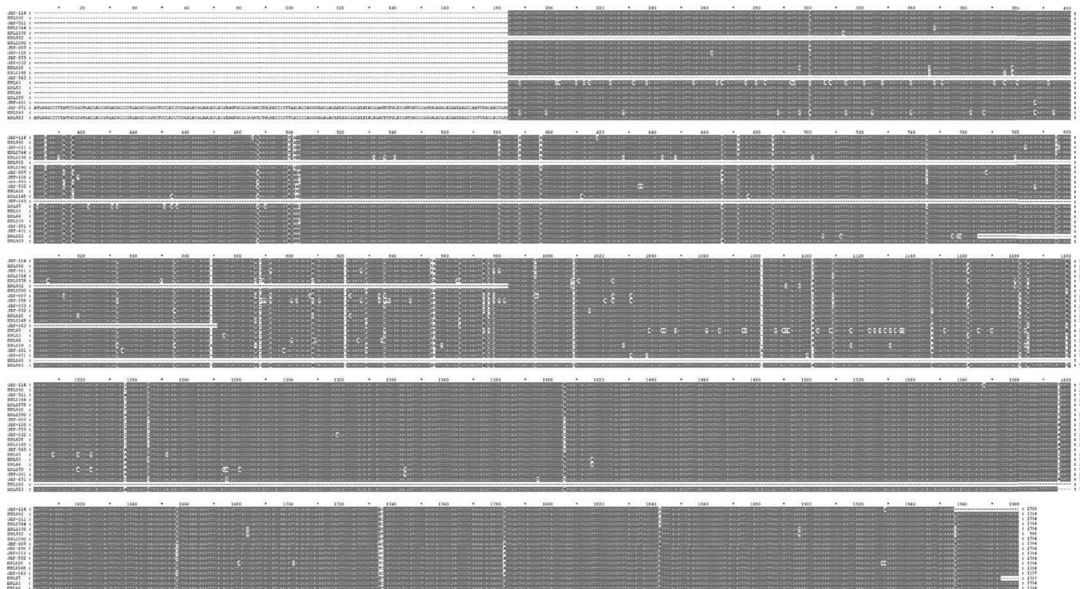

Lectin-like protein

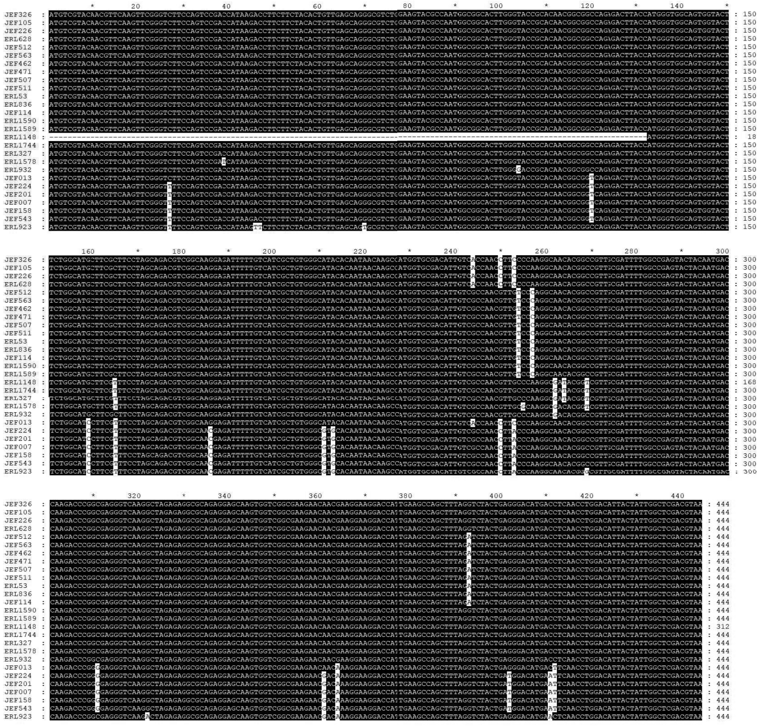

Biotrophy associated secreted preotein 2

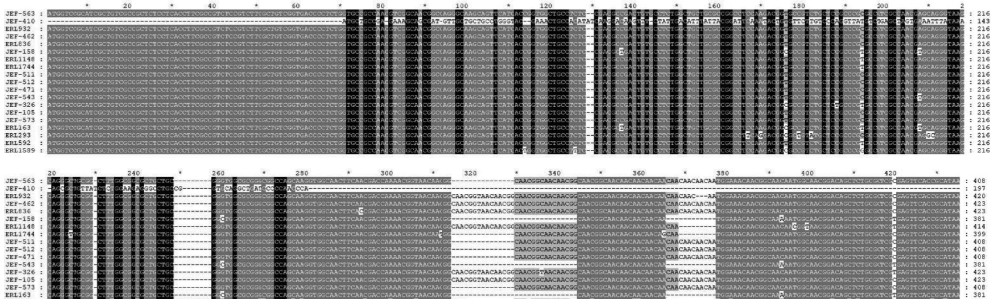

LCCL-domain containing protein

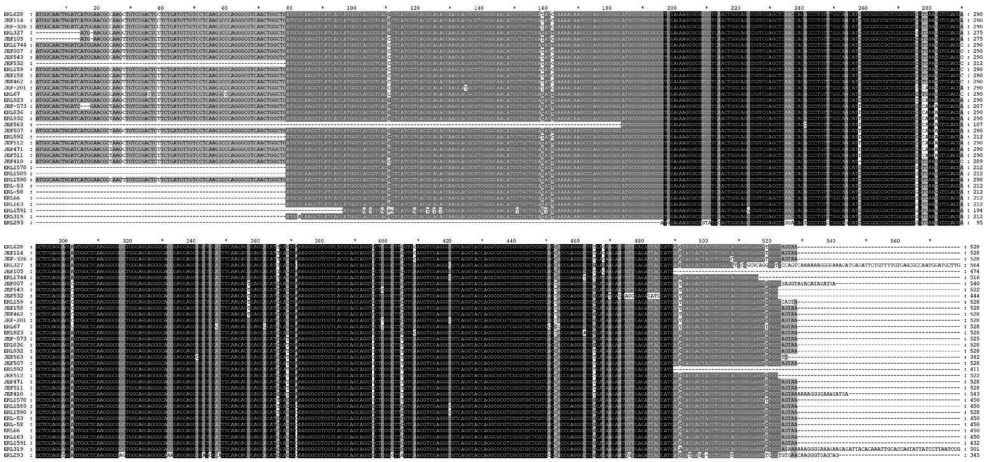

Thioredoxin-like protein

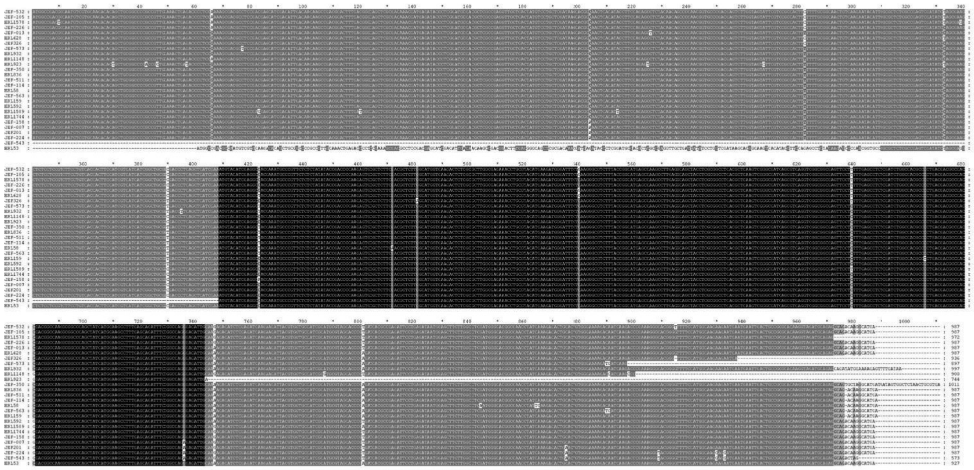

Volvatoxin A2 precursor

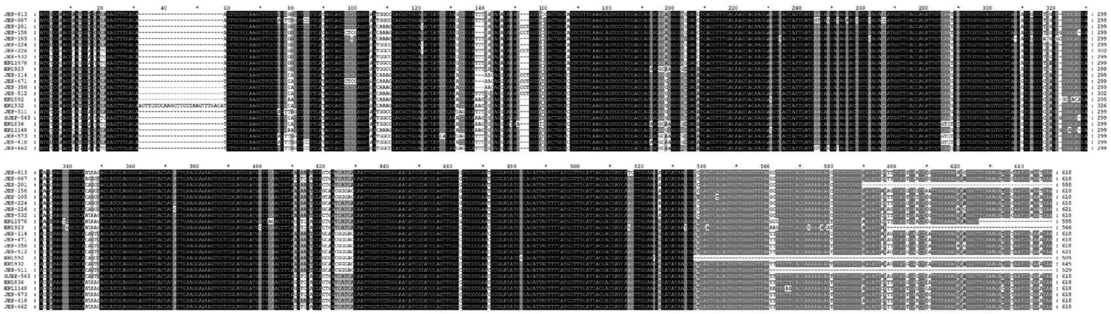

Chitinase

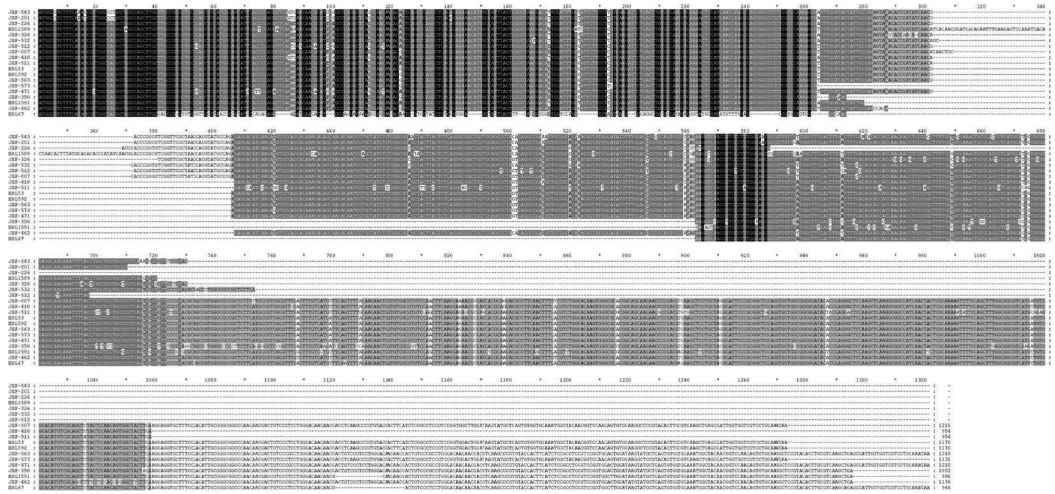

Cyclophilin B

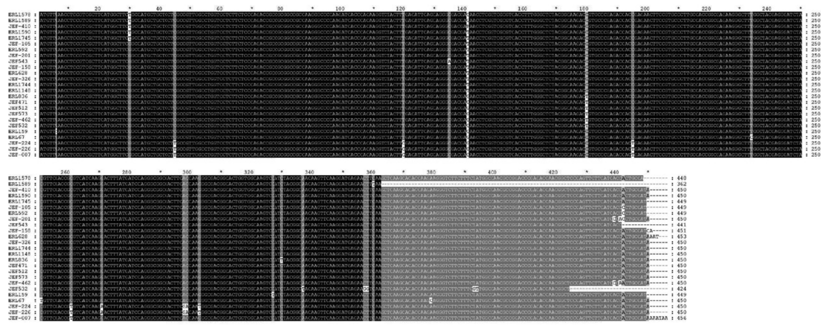

Supplementary Figure 1. Alignment of nucleotide sequences from several *B. bassiana* isolates using ClustalX. A total of nine genes were analyzed. The NCBI accession numbers of the aligned *B. bassiana* sequences are as follow. *DNA photolyase*: MK804437 to MK804467; *Lectin-like gene*: MN056915 to MN056941; *Biotrophy associated secreted protein 2*: MN225976 to MN225994; *LCCL domain containing protein*: MK852338 and MK947057 to MK947090; *Volvatoxin A2 precursor*: MN393009 to MN393031; *Thioredoxin-like protein*: MN127788 to MN127814; MSB2: MN149370 to MN149388; *Chitinase*: MN393032 to MN393050; and *Cyclophilin B*: MN182536 to MN182560.

DNA photolyase

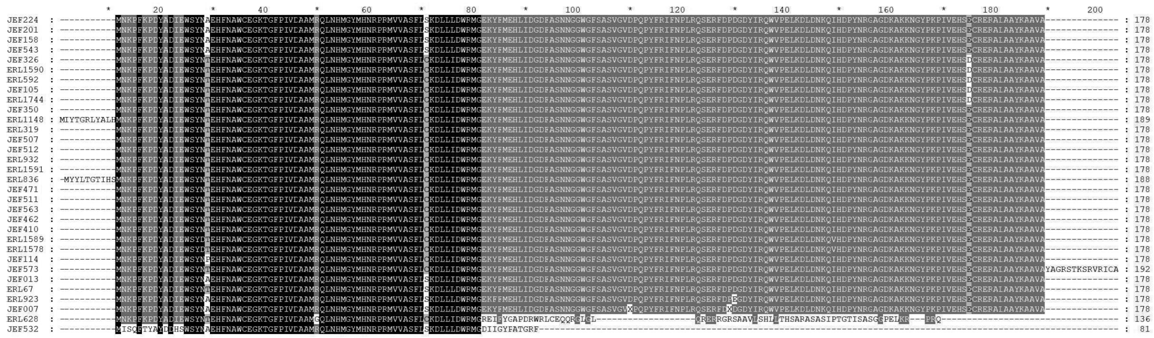

Lectin-like protein

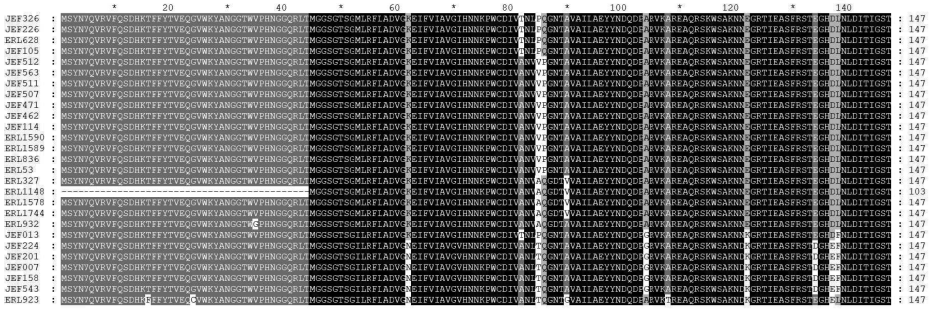

MSB2

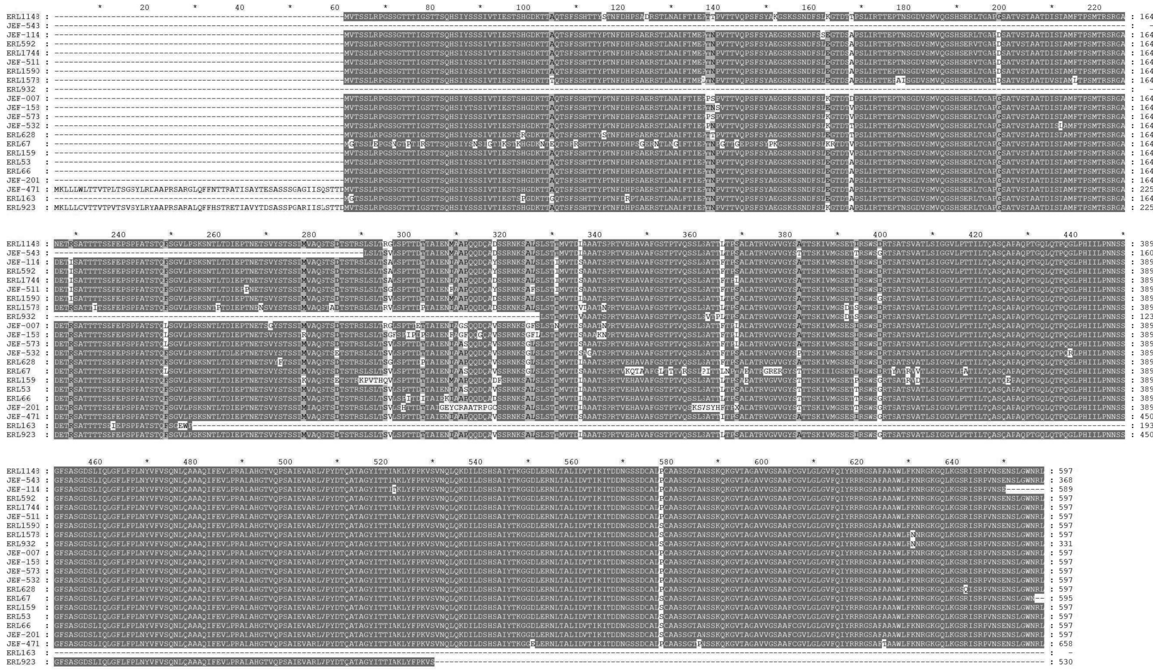

Biotrophy associated secreted preotein 2

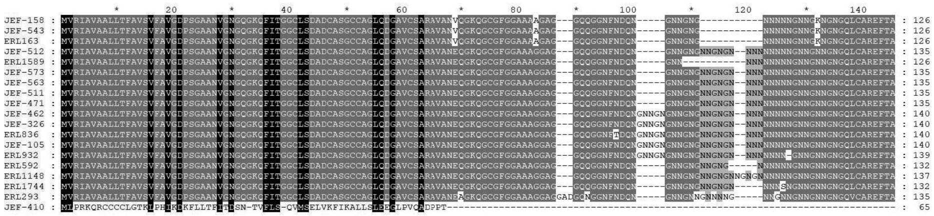

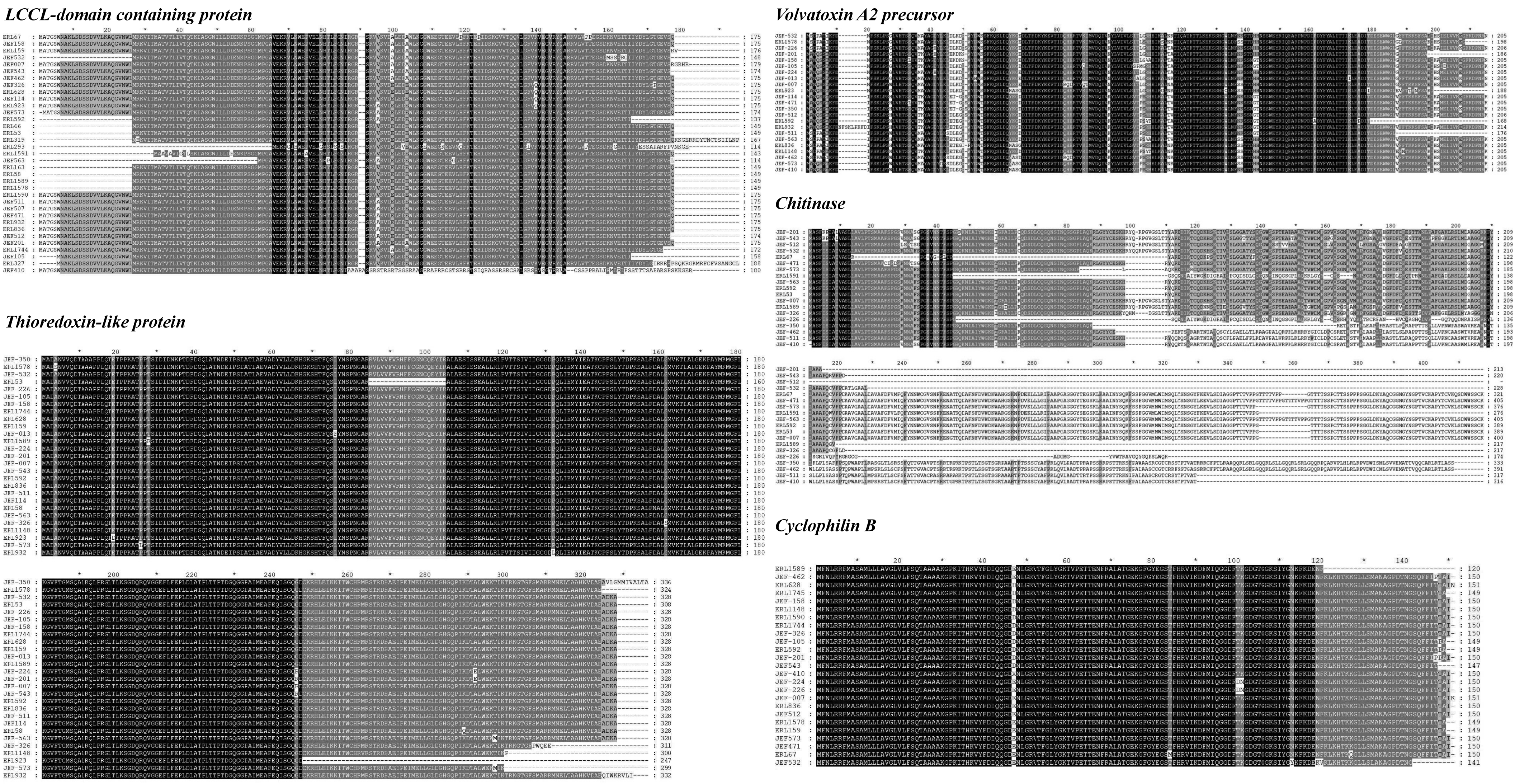
LCCL-domain containing protein
Thioredoxin-like protein
Volvatoxin A2 precursor
Chitinase
Cyclophilin B

Supplementary Figure 2. Alignment of amino acid sequences from several *B. bassiana* isolates using ClustalX. A total of nine genes were analyzed.

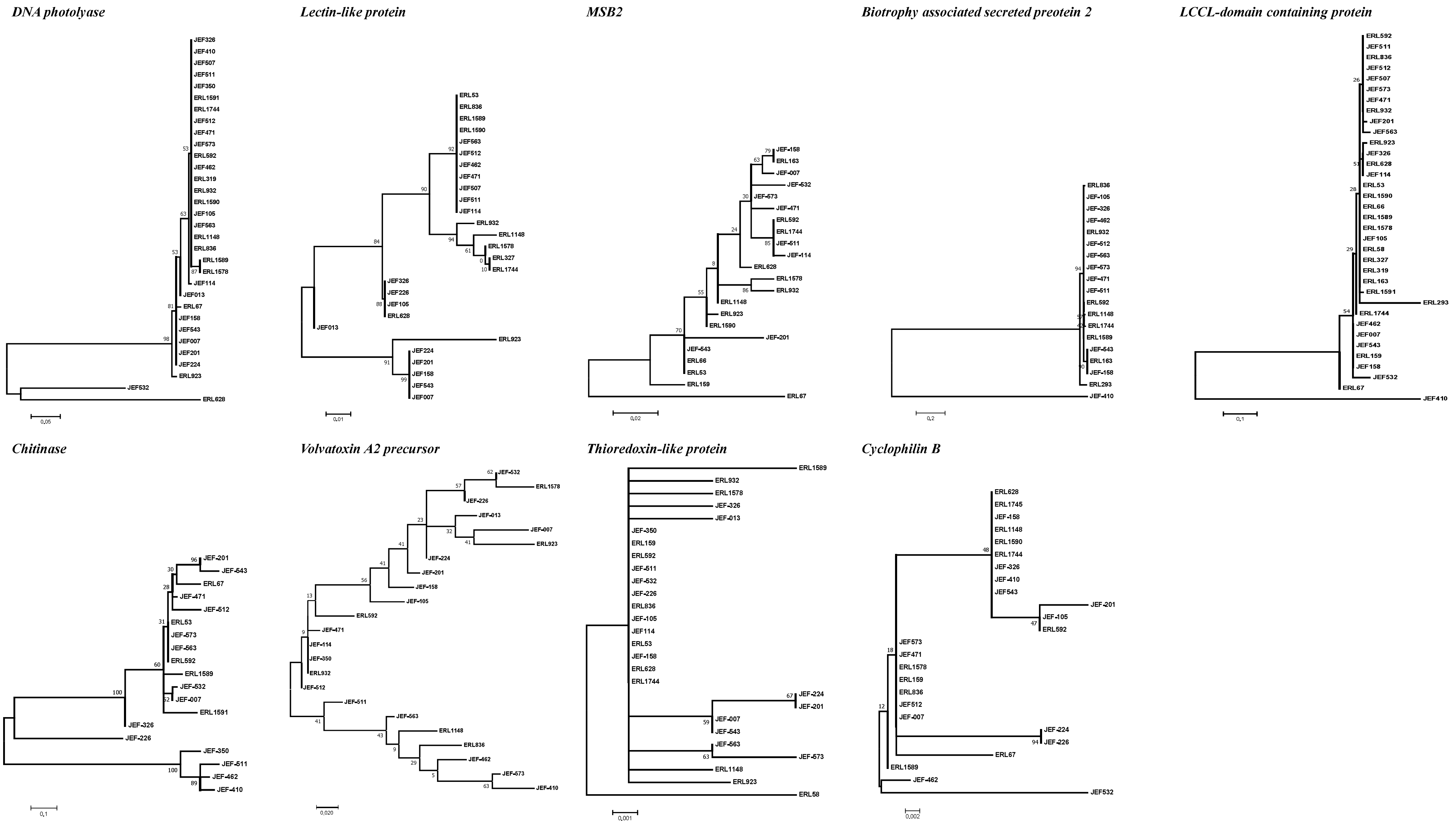
DNA photolyase
0,05
Lectin-like protein
0,01
MSB2
0,02
Biotrophy associated secreted preotein 2
0,2
LCCL-domain containing protein
0,1
Chitinase
0,1
Volvatoxin A2 precursor
0,020
Thioredoxin-like protein
0,001
Cyclophilin B
0,002

Supplementary Figure 3. Phylogenetic relationships of *B. bassiana* isolates in each gene which were analyzed using amino acid sequences. The evolutionary distance for all the trees was calculated for aligned sequences by maximum likelihood method and the numbers on the tree branches represent bootstrap values.

Supplementary table 1. List of the primers used in this study

Primer	Use	Orientation	Sequence
DNA photolyase	Amplify *DNA photolyase* contig	Forward	GAACTGTGTCTGCTGGTGGA
		Reverse	TACGTGGCGATAAAAGTCCC
Lectin-like gene	Amplify *Lectin-like gene* contig	Forward	TATACTTACTGCAACATCACT
		Reverse	GCAAGTAAAATATGAATGTTAT
Biotrophy associated gene 2	Amplify *Biotrophy associated gene 2* contig	Forward	TGGCTTGGGGGCTTCTCGCTG
		Reverse	CGGCCAGGGCAAGCAGTTCAT
LCCL-domain containing gene	Amplify *LCCL-domain containing gene* contig	Forward	CGCTCTATTTCGCAACTA
		Reverse	GAGTAGCAAGAGATGGTTCATT
Volvatoxin A2 precursor	Amplify *Volvatoxin A2 precursor* contig	Forward	CTTTATCCTTTGAGGTAGCA
		Reverse	GTAGAGAACTCCATACAACGA
Thioredoxin-like protein	Amplify *Thioredoxin-like protein* contig	Forward	GATTCTTCACATGCACGCTT
		Reverse	ATCCTAGATAATGCCCAGCT
MSB2	Amplify *MSB2* contig	Forward	CCGCGCAAAAGTCTACTTTGT
		Reverse	CACCCCTCGCTTGTGATGTT
Cyclophilin B	Amplify *Cyclophilin B* contig	Forward	AAAGGTGTCAAGTGTCCTCC
		Reverse	ATGGCAGTGGTGATGAAGAA
Chitinase	Amplify *Chitinase* contig	Forward	GTGCTAATAGTCTGGAATGCA
		Reverse	GACGCGTACCTATTTTGCC
q-*DNA photolyase*	Quantitative PCR of *DNA photolyase*	Forward	ACCGGGTTTCCCATCGTTGA
		Reverse	GCCCATGCGCCAGTCTATGA
q-*Lectin-like gene*	Quantitative PCR of *Lectin-like gene*	Forward	GAGCAGGGCGTCTGGAAGTA
		Reverse	ACCACTGCCACCCATGGTAA
q-*Biotrophy associated gene 2*	Quantitative PCR of *Biotrophy associated gene 2*	Forward	GACTGTGCCTCTGGCTGCT

		Reverse	CGTTGAAGTTGCCACCTTGCT
q-*LCCL-domain containing gene*	Quantitative PCR of *LCCL-domain containing gene*	Forward	
		Reverse	CTGACACCCTCGACCTGGAC
q-*Volvatoxin A2 precursor*	Quantitative PCR of *Volvatoxin A2 precursor*	Forward	CAAGCAGTCCCTCGACCAGT
		Reverse	GGGTGACGCTAAGCACGTC
q-*Thioredoxin-like protein*	Quantitative PCR of *Thioredoxin-like protein*	Forward	CTTCGACGCTCTTGGCATGG
		Reverse	TGCCTCAAGGCTTGGCTCAT
q-*MSB2*	Quantitative PCR of *MSB2*	Forward	TGGTCGCTCAGTCTACATCGG
		Reverse	AACAGCCTGGTCTTGTTGCG
q-*Cyclophilin B*	Quantitative PCR of *Cyclophilin B*	Forward	GGGCTTTGGCTACGAGGGAT
		Reverse	GATGGACTTGCCACCAGTGC
q-*Chitinase*	Quantitative PCR of *Chitinase*	Forward	CACTTCATCTCCGCCTCCGT
		Reverse	GGAGCCTTGCACACTGTTGG
Gamma-actin (HQ232398.1)	Quantitative PCR of the internal control	Forward	ATCCACGCCACCACCTTCAA
		Reverse	GTCGGCAAGACCAGGGTACA

Supplementary table 2. Selection pressures exerted on the different studied genes. In the column corresponding to aBSREL analyses, + denote for evidence for positive diversifying selection pressures and 0 denote for neutral selection. In the columns corresponding to FUBAR, MEME and SLAC analyses, numbers refer to number of codons found to be subjected to selection pressures while + and –refer to positive or diversifying selection and negative or purifying selection respectively. The number of codons in front of each gene name represent the number of codons or amino acids considered for each gene in the codon based analyses.

**Gene**	**Gene analyses**	**Codon based analyses**		
	**aBSREL**	**FUBAR**	**MEME**	**SLAC**
DNA photolyase (180 codons)	+	1 +<br>9 -	1	0 +<br>1 -
LCCL-domain containing protein (176 codons)	+	0 +<br>16 -	2	2+<br>1 -
Volvatoxin A2 precursor (206 codons)	+	11 +<br>11 -	16	1+<br>10 -
Lectin-like gene (147 codons)	0	0 +<br>2 –	1	0 +<br>0 -
Biotrophy associated secreted protein 2 (136)	+	0 +<br>3 -	2	0 +<br>2 -
MSB2 (598 codons)	+	7 +<br>6 -	9	0 +<br>6 -
Theoredoxin-like protein (149 codons)	+	0 +<br>11 -	7	0 +<br>9 -
Chitinase (405 codons)	+	0 +<br>5 -	3	3 +<br>0 -
Cyclophilin B	0	0+<br>0 -	0	0 +<br>0 -

Supplementary table 3. Diversifying positive selection pressures exerted on the different isolates. + and – refer to evidence and no evidence for selection pressures respectively, and ND refers to non determined due to non amplification of the gene from the corresponding isolates. The shaded lines of the table show the isolates that are subjected to selection pressures at 2 or more genes among the 9 studied genes.

	DNA photol-yase	*LCCL-domain containing protein*	*Volvatoxin A2 precursor*	*Biotrophy associated secreted protein 2*	*Lectin-like protein*	*MSB 2*	*Theoredoxin-like protein*	*Chitinase*	*Cyclophilin B*
**JEF-007**	-	-	-	ND	-	-	-	+	-
**JEF-013**	-	ND	+	ND	-	-	-	ND	ND
**JEF-105**	-	-	-	-	-	-	-	ND	-
**Jef-114**	-	-	-	ND	-	-	-	ND	ND
**JEF-158**	-	-	+	-	-	-	-	ND	-
**JEF-162**	ND	ND	ND	ND	ND	-	ND	ND	ND
**JEF-201**	-	-	+	ND	-	-	-	+	-
**JEF-224**	-	ND	-	ND	-	-	-	ND	-
**JEF-226**	ND	ND	-	ND	-	-	-	+	-
**JEF-326**	-	-	ND	+	-	-	+	-	-
**JEF-350**	-	ND	+	ND	ND	-	-	+	ND
**JEF-410**	-	-	+	-	ND	-	ND	+	-
**JEF-462**	-	-	-	-	-	-	ND	+	-
**JEF-471**	-	-	+	+	-	-	ND	-	-
**JEF-507**	-	-	ND	ND	-	-	ND	ND	ND
**JEF-511**	-	-	-	+	-	-	-	+	ND
**JEF-512**	-	-	-	+	-	-	ND	-	-
**JEF-532**	+	+	-	ND	ND	-	-	+	-
**JEF-543**	-	-	ND	+	-	-	+	-	-
**JEF-563**	-	-	+	-	-	-	-	-	ND
**JEF-573**	-	-	-	-	ND	-	-	+	-
**ERL53**	ND	-	ND	ND		-	+	-	ND
**ERL58**	ND	-	ND	ND	ND	ND	-	ND	ND
**ERL66**	ND	-	ND	ND	ND	-	ND	ND	ND
**ERL67**	-	-	ND	ND	ND	-	ND	+	-
**ERL159**	ND	-	ND	ND	ND	+	-	ND	-
**ERL163**	ND	-	ND	-	ND	-	ND	ND	ND
**ERL293**	ND	-	ND	-	ND	ND	ND	ND	ND
**ERL319**	-	-	ND	ND	ND	ND	ND	ND	ND
**ERL327**	ND	-	ND	ND	-	ND	ND	ND	ND
**ERL592**	-	-	-	-	-	-	-	-	-
**ERL628**	+	-	ND	ND	-	-	-	ND	-
**ERL836**	+	-	+	+	-	ND	-	ND	-
**ERL923**	-	-	-	+	-	-	-	ND	ND
**ERL932**	-	-	+	+	-	-	+	+	ND
**ERL1148**	-	-	+	-	-	-	-	ND	-
**ERL1578**	-	-	-	ND	-	-	-	ND	-
**ERL1589**	-	-	ND	-	-	ND	-	+	-
**ERL1590**	-	-	ND	ND	-	-	ND	ND	ND
**ERL1591**	-	-	ND	ND	ND	ND	ND	+	ND
**ERL1744**	-	-	ND	+	-	-	-	ND	-
**ERL1745**	ND	ND	ND	ND	ND	ND	ND	ND	-

Supplementary table 4. Virulence of the 42 selected isolates against the mealworm *Tenebrio molitor* and the silverleaf whitefly *Bemicia tabaci*.

**Isolates**	**Mortality percentage of *T. molitor* larvae 10 days after treatment (Mean ± SE)**	**Mortality percentage of *B. tabaci* larvae 5 days after treatment (Mean ± SE)**
JEF-007	63.3 ±8.8	79.4 ± 4.9
JEF-013	60.0 ± 8.9	60 ± 5.7
JEF-105	36.6 ± 8.8	59.2 ± 5.6
JEF-114	50.0 ± 9.1	72.5 ± 7.1
JEF-158	56.7 ± 9.1	79.6 ± 6.1
JEF-162	60.0 ± 8.9	69.4 ± 6.6
JEF-201	57.7 ± 9.7	64.4 ± 6.2
JEF-224	76.7 ± 7.7	52.6 ± 6.6
JEF-226	60 ± 8.9	40.4 ± 7.2
JEF-326	80 ± 7.3	79.3 ± 5.3
JEF-350	83.3 ± 6.8	83.9 ± 4.7
JEF-410	86.7 ± 6.2	69.7 ± 5.4
JEF-462	90 ± 5.5	84.9 ± 4.4
JEF-471	79.3 ± 7.5	62.5 ± 6.1
JEF-507	90 ± 5.5	74.4 ± 4.8
JEF-511	80 ± 7.3	70.3 ± 5.7
JEF-512	80 ± 7.3	66.7 ± 6.2
JEF-532	79.3 ± 7.5	74.4 ± 4.8
JEF-543	81.3 ± 6.9	68.6 ± 6.5
JEF-563	80 ± 7.3	59.5 ± 5.7
JEF-573	80 ± 7.3	60.7 ± 6.5
ERL53	36.7 ± 8.8	50 ± 6.7
ERL58	28.1 ± 8.6	52.3 ± 5.3
ERL66	36.7 ± 8.8	45.1 ± 7
ERL67	66.7 ± 8.6	60.3 ± 6.2
ERL159	30 ± 8.4	45.3 ± 5.8
ERL163	66.7 ± 8.6	42.9 ± 6.6
ERL293	61.3 ± 8.8	75 ± 6.3
ERL319	26.7 ± 8.1	64.2 ± 6.6
ERL327	31.0 ± 8.6	69.2 ± 6.4
ERL592	43.3 ± 9.1	71.8 ± 7.2
ERL628	83.3 ± 6.8	70.8 ± 5.4
ERL836	80 ± 7.3	86.8 ± 4.7
ERL923	86.7 ± 6.2	83.1 ± 4.9
ERL932	76.7 ± 7.7	66.7 ± 6.4
ERL1148	12.9 ± 6.0	59.7 ± 6.9
ERL1578	66.7 ± 8.6	80 ± 4.6
ERL1589	46.7 ± 9.1	59.4 ± 6.1
ERL1590	3.3 ± 3.3	65.5 ± 6.4
ERL1591	13.3 ± 6.2	71.4 ± 6.0
ERL1744	20 ± 7.3	79.3 ± 5.6
ERL1745	80 ± 7.3	48.7 ± 8

Supplementary table 5. Summary of the different performed correlation analyses between genetic diversity and biological performance. – and + symbols refer to negative correlation and positive correlation, respectively; and NC refers to no correlation

Gene	Non-synonymous change (NSC)				Copy number variation (CVN)			
	**Virulence: *T. molitor***	**Virulence: *B.tabaci***	**Growth rate**	**Thermo -tolerance**	**Virulence: *T. molitor***	**Virulence: *B.tabaci***	**Growth rate**	**Thermo -tolerance**
Biotrophy associated protein 2	NC	NC	NC	-	+	NC	NC	+
Chitinase	NC	+	+	NC	-	NC	NC	-
Cyclophilin B	NC	NC	NC	NC	NC	-	NC	NC
DNA photolyase	NC	NC	NC	NC	NC	NC	NC	-
Lectin-like protein	-	NC	NC	-	NC	NC	NC	-
LCCL- domain containing protein	NC	NC	NC	NC	NC	NC	NC	NC
MSB2	+	NC	NC	NC	NC	-	NC	NC
Thioredoxin-like protein	NC	NC	-	NC	NC	NC	NC	NC
Volvatoxin A2 precursor	NC	-	NC	NC	NC	NC	NC	NC
